# Fetal and infant origins of asthma

**DOI:** 10.1007/s10654-012-9657-y

**Published:** 2012-02-18

**Authors:** Liesbeth Duijts

**Affiliations:** 1 Department of Epidemiology, Erasmus Medical Center, Rotterdam, The Netherlands; 2 Department of Epidemiology and Pediatrics—division of Respiratory Medicine, Erasmus Medical Center, Rotterdam, The Netherlands; 3 Division of Respiratory Medicine (Sp-3435), Department of Pediatrics, Erasmus Medical Center, PO Box 2060, 3000 CB Rotterdam, The Netherlands

**Keywords:** Cohort study, Foetus, Infant, Asthma, Low birth weight, Preterm birth, Growth, Smoke exposure, Maternal diet, Breastfeeding, Respiratory tract infections, Acetaminophen, Gene, Epigenetics

## Abstract

Previous studies have suggested that asthma, like other common diseases, has at least part of its origin early in life. Low birth weight has been shown to be associated with increased risks of asthma, chronic obstructive airway disease, and impaired lung function in adults, and increased risks of respiratory symptoms in early childhood. The developmental plasticity hypothesis suggests that the associations between low birth weight and diseases in later life are explained by adaptation mechanisms in fetal life and infancy in response to various adverse exposures. Various pathways leading from adverse fetal and infant exposures to growth adaptations and respiratory health outcomes have been studied, including fetal and early infant growth patterns, maternal smoking and diet, children’s diet, respiratory tract infections and acetaminophen use, and genetic susceptibility. Still, the specific adverse exposures in fetal and early postnatal life leading to respiratory disease in adult life are not yet fully understood. Current studies suggest that both environmental and genetic factors in various periods of life, and their epigenetic mechanisms may underlie the complex associations of low birth weight with respiratory disease in later life. New well-designed epidemiological studies are needed to identify the specific underlying mechanisms. This review is focused on specific adverse fetal and infant growth patterns and exposures, genetic susceptibility, possible respiratory adaptations and perspectives for new studies.

Asthma in childhood has a high prevalence across many countries worldwide [1]. The reported prevalence of asthma is around 5–10% among school-age children and figures are even higher for asthma related symptoms, such as wheezing, in younger children. Childhood asthma is related to a reduced quality of life and exercise tolerance, and higher risks of school absenteeism and hospitalization [2]. Despite the availability of effective treatments for symptoms, the morbidity remains high [3]. The lack of curative options seems to be largely due to the unknown aetiology of asthma [4]. Furthermore, one general definition of asthma is difficult to define [5]. Objective tests, including spirometry or bronchial hyperresponsiveness, are not easy to conduct in young children, and have limited applicability. In epidemiological studies it is currently well accepted that a diagnosis of asthma is based on parental- or self-reported symptoms [6]. Because of these different asthma definitions, it is important to identify the specific underlying mechanisms for the associations of exposures in fetal life and infancy with different asthma related outcomes, which might reflect different specific structural and functional adaptations.

Several studies, of which some have been published in the *European Journal of Epidemiology* have suggested that asthma, like other common diseases, has at least part of its origin early in life [4, 7–41]. Long term follow up studies in different populations have shown that impaired respiratory health or lung function in early childhood is associated with asthma and other respiratory diseases in later life [42–48]. These studies suggest that lung function and susceptibility for various respiratory diseases track from early childhood onwards. Thus, risk factors for wheezing and asthma or low airway function in childhood might predispose for respiratory diseases in later life. Examples of known major risk factors in early childhood for development of respiratory disease or low airway function include exposure to parental smoking or air pollution, no or shorter period of breastfeeding, obesity, larger family size, daycare attendance, infectious diseases in early childhood and acetaminophen use [49–56].

## Developmental origins of childhood asthma

Recently, low birth weight has also been shown to be associated with increased risks of asthma, chronic obstructive airway disease, and impaired lung function in adults [57–61]. In term born children, it was found that low birth weight is associated with increased risks of respiratory symptoms in the first 7 years of life [62]. Low birth weight per se is not likely to be the causal factor leading to asthma. The same birth weight might be the result of various growth patterns and different fetal exposures [10, 63]. The developmental plasticity hypothesis suggests that the associations between low birth weight and diseases in later life are explained by early adaptation mechanisms in response to various adverse exposures in fetal life and early childhood [64]. This hypothesis is supported by extensive epidemiological evidence showing strong and consistent associations of low birth weight with the risk of common diseases in adulthood, including cardiovascular disease, type 2 diabetes and COPD [10, 64–66]. Developmental adaptations in fetal life and infancy might also result in impaired lung growth, leading to smaller airways, decreased lung volume, and subsequently to an increased risk of asthma or COPD throughout postnatal life [46, 58, 60, 66, 67]. Reduced diameter of central and small airways can contribute to the development of childhood asthma [68–70]. Airway caliber is a key determinant of total airway resistance and might be related to asthma and COPD [71]. Other mechanisms underlying the associations of low birth weight with asthma and respiratory diseases in childhood and adulthood might include an innate or T helper 2 skewed immune system, increased allergen sensitization, inflammation and bronchial hyperreactivity [72–75]. These different underlying mechanisms may lead to various phenotypes of asthma with onset at different ages.

## Fetal growth characteristics

Studies with information about fetal growth characteristics in different periods of pregnancy enable identification of critical fetal periods that might be important for the risk of asthma and other respiratory diseases [76, 77]. A recent study suggested no associations of fetal growth characteristics with the risk of ‘ever wheeze’ until the age of 3 years [78]. However, when the outcome wheeze was combined with atopic status of the child, the authors showed that a SD lower fetal head circumference growth between 11 and 19 weeks was associated with a 10% higher risk of non-atopic wheeze, and a SD lower fetal abdominal circumference growth between 19 and 34 weeks of gestation was associated with a 20% higher risk of atopic wheezing. Another study with retrospectively collected fetal measurements observed that reduced fetal size in first trimester is associated with increased risks for asthma and airway obstruction in children aged 10 years [79]. This study did not take postnatal growth parameters into account. However, infant weight gain, which is generally higher in children with a low birth weight, is also associated with respiratory morbidity in childhood [78, 80]. Recently, a prospective population-based cohort study among 5,125 children observed that not fetal growth, but weight gain acceleration in early infancy was associated with increased risks of asthma symptoms in preschool children, independent of fetal growth [81]. These results suggest that early infancy might be a more critical period for the development of asthma. The associations of longitudinally measured fetal and early childhood growth patterns with various phenotypes of asthma, lung function and lung structure in childhood and later life adjusted for potential confounders remain to be studied in further detail.

In addition to fetal and early childhood growth, preterm birth is suggested to be associated with asthma and impaired lung function [81–88]. A recent meta-analysis showed that preterm birth defined as a gestational age less than 37 weeks at birth was associated with an 1.07–1.36 increased risk of asthma, depending on the heterogeneity of the studies, compared with children born at term [82]. This effect seemed stronger at a younger age but this difference might reflect differences between study populations rather than a real age effect. Potential underlying pathways for the association between preterm birth and asthma are not disentangled yet but might include underdeveloped anatomical or immunological mechanisms [89, 90], interaction with environmental factors, such as smoke exposure and chorioamnionitis [49, 84], or genetic factors [91]. Also, the degree of immaturity, therapeutic interventions and co-morbidity might play a role. Recent studies observed inconsistent results on the associations of gestational age and risks of asthma for children born late preterm [85, 87, 88] and children born early preterm with or without mechanical ventilation [88] or bronchopulmonary dysplasia [86, 92]. Future studies are needed to elaborate on these aspects.

## Maternal smoking and diet

Maternal smoking is the most important adverse fetal exposure in Western countries, and is strongly associated with fetal growth retardation and low birth weight [63]. Recent studies have demonstrated that maternal smoking during pregnancy is associated with increased risks of wheezing and asthma during childhood [93–99]. Most studies were not able to assess the effect of maternal smoking exposure in different periods of pregnancy, which might be important for identifying critical periods. It is also not known whether the associations between maternal smoking during pregnancy and the risk of childhood asthma are explained by direct intrauterine effects, or reflect other unmeasured environmental confounders. Stronger effect estimates for maternal smoking than for paternal smoking during pregnancy with childhood asthma would suggest direct intra-uterine effects, whereas similar effect estimates suggest that the associations are explained by unmeasured socio-economic, or life style related factors [100, 101]. Also, as in all observational designs, residual confounding might still be an issue due to unmeasured social and life style related factors. This needs to be explored in detail. Recently, we have demonstrated that continued maternal smoking throughout pregnancy is associated with asthma symptoms, including wheezing, in preschool children [49]. These associations were independent of paternal smoking and imply a direct adverse effect of smoke exposure on fetal lung development. The mechanisms by which maternal smoking during pregnancy affect body and lung growth are not fully understood, but may include direct toxic effects on the respiratory system and DNA methylation of genes. Recent studies showed general and biological pathway specific differences in DNA methylation patterns in children from mothers who smoked during pregnancy [102, 103]. Differences in DNA-methylation within the promoter regions of particular genes may alter the expression of the gene products related to growth and lung development. However, it is not known whether the associations between low birth weight and respiratory disease can be fully explained by fetal smoke exposure, and the related changes in DNA methylation.

Suboptimal fetal nutrition, due to maternal obesity or underweight, insufficient dietary intake, or placental dysfunction might also affect fetal growth and lung development [52, 104–107]. Maternal obesity and placenta dysfunction are strongly related to fetal growth, but their direct associations with respiratory health in the offspring are not fully understood yet [10, 108]. Insufficient maternal dietary intake of macronutrients during pregnancy may lead to impaired fetal body, lung and airway growth and subsequently to an increased risk of asthma in childhood [104, 109–116]. More specifically, maternal Mediterranean and Western dietary patterns during pregnancy are related to fetal growth patterns [117, 118]. Thus far, little is known whether these maternal dietary patterns also affect the risk of childhood asthma [119]. The mechanisms by which dietary patterns affect body and lung growth development may also include DNA methylation [120, 121]. Lower intake of micronutrients such as folate, and vitamin B12 in mother’s diet may induce epigenetic changes, since folate and vitamin B12 are important methyl donors during pregnancy [122]. Vitamin E has the potential to influence airway development via epigenetic mechanisms because it influences gene expression and airway epithelial cell signalling [112]. The role of epigenetic mechanisms regarding the association of low maternal vitamin D intake during pregnancy with a higher incidence of asthma and wheeze in children is not known yet [123–127]. The importance of the maternal intake of these micronutrients on respiratory health in childhood and adulthood might differ between developing and Western countries, since the nutritional status of mothers and children in these countries are different.

## Exposures in infancy

After birth, infant diet may also influence body and lung growth development [128]. Specific infant feeding patterns such as early introduction of bottle feeding or solid food instead of a long period of exclusive breastfeeding may lead to reduced lung and airway growth and increased risk of childhood asthma [50, 129–132]. Underlying mechanisms that have been suggested to explain the associations of shorter duration or smaller amount of breastfeeding with the risks of asthma are breast milk components, including IgA, cytokines, glycans and long-chain fatty acids that stimulate and balance the infant’s innate immune system and growth [133–135]. Atopic and infectious mechanisms might partly mediate the protective effect of breastfeeding on childhood asthma [50, 131, 136, 137]. Thus far, previous studies did report inconsistent results on effect modification by family history of asthma, allergy and atopy on the association of breastfeeding with wheezing [131, 136–139]. Also, the protective effect of a longer period of breastfeeding duration on childhood asthma still remains when respiratory tract infections are taken into account [50]. Further studies are needed to explore in detail the role of atopy, infections, suboptimal lung growth [128], epigenetic changes [140] and other possible underlying mechanisms in the associations between breastfeeding and asthma.

In the first years, childhood asthma symptoms or wheezing are predominantly related to respiratory tract infections [53, 55, 56, 141, 142]. Respiratory infectious diseases in infancy also predict the risk of asthma and other respiratory diseases in childhood and adulthood [66, 143, 144]. Results from the COPSAC study have shown that colonization of the airways with *S*. *pneumoniae*, *H*. *influenzae*, *M*. *catarrhalis*, or more than one of these organisms in asymptomatic neonates at 1 month of age was associated with increased risks of wheezing symptoms, higher blood eosinophil counts and total IgE, and development of asthma in school age children [56]. Similarly, nasal carriage with *S*. *aureus* is associated with an increased risk of atopic dermatitis [145]. Still, whether these associations reflect causal mechanisms or reflect symptoms of the same underlying susceptible lung or skin is not known. Acetaminophen is frequently used in children with fever episodes, including those with upper and lower respiratory tract infections. Several studies have suggested associations between acetaminophen in childhood and the risk of asthma [54, 146–150]. A recent review summarized the existing evidence for the associations of acetaminophen use with childhood asthma, and suggested that this association might be causal based on various epidemiological findings including the strength, consistency, and dose–response relationship [151]. Also, the metabolism of acetaminophen leads to a depletion in airway mucosal glutathione that could contribute to vulnerability to oxidant stress. Prospective studies relating infectious diseases and acetaminophen use with different respiratory health outcomes in adulthood have not been performed yet.

## Genetic susceptibility

The associations of low birth weight with respiratory symptoms in later life might also be explained by common genetic variants leading to both fetal growth restriction and smaller lungs and airways and, subsequently, to asthma. Previous candidate gene studies and linkage studies identified more than 100 genes associated with asthma [152, 153]. However, most of these associations could not be replicated. More recent genome wide approaches in large study populations successfully identified and replicated genetic variants related to asthma in children [154–160]. Variants in the *ORM1*-*like 3* (*ORMDL3*) gene and in neighbouring genes, such as gasdermin A and B (*GSDM*), have consistently been associated with risk of childhood asthma. None of the identified common genetic variants related to asthma or COPD were associated with birth weight on a genome wide significant level [161], but this might be due to lack of power. Thus far, no genome wide association studies have been performed on lung function in early childhood. Therefore, further studies are needed that relate genetic variants to both early body and lung growth characteristics and respiratory diseases in later life.

The effects of early life exposures such as maternal smoking and diet, breastfeeding and infant infectious diseases on the risk of asthma and COPD might be modified by a genetic susceptibility. Recent studies suggest that the effects of some genetic variants related to asthma, located on *glutathione S*-*transferase* (*GST)*, *methylenetetrahydrofolate reductase* (*MTHFR*), *gasdermin*-*like* (*GSDML*) and *ORMDL3* genes, are modified by environmental exposures, including tobacco smoke exposure [116, 162–167]. The increased risk of early-onset asthma due to 17q21 genetic variants of *ORDML3*, *GSDML* and 2 other genes was further enhanced by early childhood tobacco smoke exposure [162, 164]. Also, *GST* genes in combination with fetal smoke exposure seem to be associated with lower airway responsiveness, lung function and increased risk of transient wheezing, a phenotype of childhood asthma [163, 166]. However, results for effect modification by maternal smoking on the associations of *GST* genes with respiratory morbidity are not consistent [167, 168]. Further gene-environment studies are needed, especially focused on the environmental exposures in the fetal period, during which period alveolar and airway development is largely completed.

## Conclusion

**Fig. 1:**
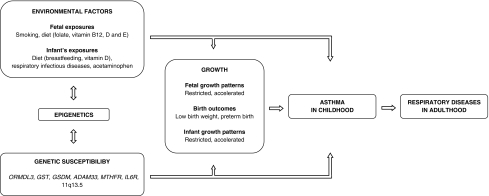
Pathways leading from adverse fetal and infant exposures to growth adaptations and respiratory health outcomes

Asthma in childhood is a heterogeneous disease and the exact underlying origins remain partly unknown. Several lines of investigation suggest that asthma has at least part of its origin in fetal life and infancy. Low birth weight seems to be associated with the risk of childhood asthma, but may not be the causal factor per se. Therefore, focus for further research should be beyond birth to identify specific fetal and infant growth patterns, their specific exposures and gene-environment interactions leading to asthma and other respiratory diseases in later life (Fig. 1). Specific early exposures of interest include maternal smoking and diet, infant feeding, and infectious diseases in early life. Developmental adaptation mechanisms in early life that need to be studied in epidemiological study designs include DNA methylation, and detailed imaging and functional assessments of the airways and lungs. Newly identified contributing factors to the origins of respiratory diseases including childhood asthma can ultimately contribute to the development of novel preventive, diagnostic and therapeutic approaches.
